# Vegetarian diets in female athletes: performance, health outcomes, and the implementation gap

**DOI:** 10.3389/fnut.2026.1901139

**Published:** 2026-08-05

**Authors:** Miguel López-Moreno, Nuria Perez-Diaz-Del-Campo, Nora Villa, Paulina Maria Leszczyńska, Jorge Sánchez-Infante, Paula Marrero-Fernández

**Affiliations:** 1 Diet, Planetary Health and Performance, Faculty of Health Sciences, Universidad Francisco de Vitoria, Pozuelo de Alarcón, Spain; 2 Institute of Health and Sport Sciences, Faculty of Health Sciences, Universidad Francisco de Vitoria, Madrid, Spain; 3 Toledo Physiotherapy Research Group (GIFTO), Faculty of Physiotherapy and Nursing of Toledo, Universidad de Castilla-La Mancha, Toledo, Spain; 4 Department of Chemical Engineering and Pharmaceutical Technology, University of La Laguna, Santa Cruz de Tenerife, Spain

**Keywords:** low energy availability, menstrual function, relative energy deficiency in sport, sports nutrition, vegetarian diet

## Abstract

Vegetarian diets have gained increasing popularity among athletes, yet evidence regarding their effects in female athletes remains limited. Concerns persist regarding performance, muscle adaptations, hormonal health, and the risk of low energy availability (LEA) and relative energy deficiency in sport (REDs). This narrative review critically evaluates the available evidence regarding vegetarian diets in female athletes, with particular emphasis on health, performance, and physiological outcomes, and the role of dietary implementation in shaping current findings. Available intervention and observational studies generally report comparable performance, physiological, and health-related outcomes between vegetarian and omnivorous diets in female athletes. Accordingly, current evidence does not indicate that vegetarian diets inherently compromise exercise performance, muscular adaptations, hormonal health, or increase REDs risk when nutritional requirements are achieved. However, the evidence base remains limited and heterogeneous. Much of the variability observed across studies may be influenced by implementation-related factors, including energy availability, risk of LEA, protein adequacy, micronutrient intake, dietary quality, and duration of dietary adherence, rather than by dietary classification alone. Collectively, the available literature suggests that health and performance outcomes in female athletes are influenced more by energy availability, nutritional adequacy, and overall dietary implementation than by the exclusion of animal-source foods *per se*. Future research should improve the characterization of implementation-related factors to support evidence-based recommendations tailored to female athletes.

## 1 Introduction

Vegetarian diets comprise a group of dietary approaches that exclude meat and meat-derived foods while differing in the extent to which other animal-source foods are included. As proposed by Hargreaves et al. (1), this umbrella term encompasses four diet types: lacto-ovo-vegetarian, lacto-vegetarian, ovo-vegetarian, and vegan diets (Table 1). They have received growing attention in recent years, driven by ethical, environmental, and health-related considerations (2, 3). This trend may also be reflected in athletic populations, particularly among endurance athletes (4), although longitudinal data on changes in their prevalence are limited (5–7). Notably, women appear to be more likely than men to adopt vegetarian diets (8–10), making female athletes a particularly relevant population in which to examine the physiological and performance-related implications of these dietary approaches.

**Table 1:** Classification of vegetarian diet types.

Diet type	Definition
Lacto-ovo-vegetarian	Excludes all meat, poultry, fish and seafood, while including dairy products and eggs.
Lacto-vegetarian	Excludes all meat, poultry, fish, seafood and eggs, while including dairy products.
Ovo-vegetarian	Excludes all meat, poultry, fish, seafood and dairy products, while including eggs.
Vegan	Excludes all foods of animal origin.

Despite growing interest in vegetarian diets among athletes, much of the available evidence has been derived from studies conducted predominantly in men or in mixed-sex populations without sex-specific analyses (11–15). This represents an important limitation because female athletes exhibit sex-specific physiological characteristics that may influence nutritional requirements, exercise metabolism, training adaptations, and health outcomes (16, 17). Consequently, findings derived from studies conducted predominantly in men cannot be directly extrapolated to female athletes. Furthermore, factors such as menstrual cycle fluctuations, hormonal contraceptive use, reproductive function and a greater susceptibility to low energy availability (LEA) may also influence both the physiological responses to dietary interventions and the practical challenges associated with implementing vegetarian diets in athletic settings (18, 19).

**Figure 1:**
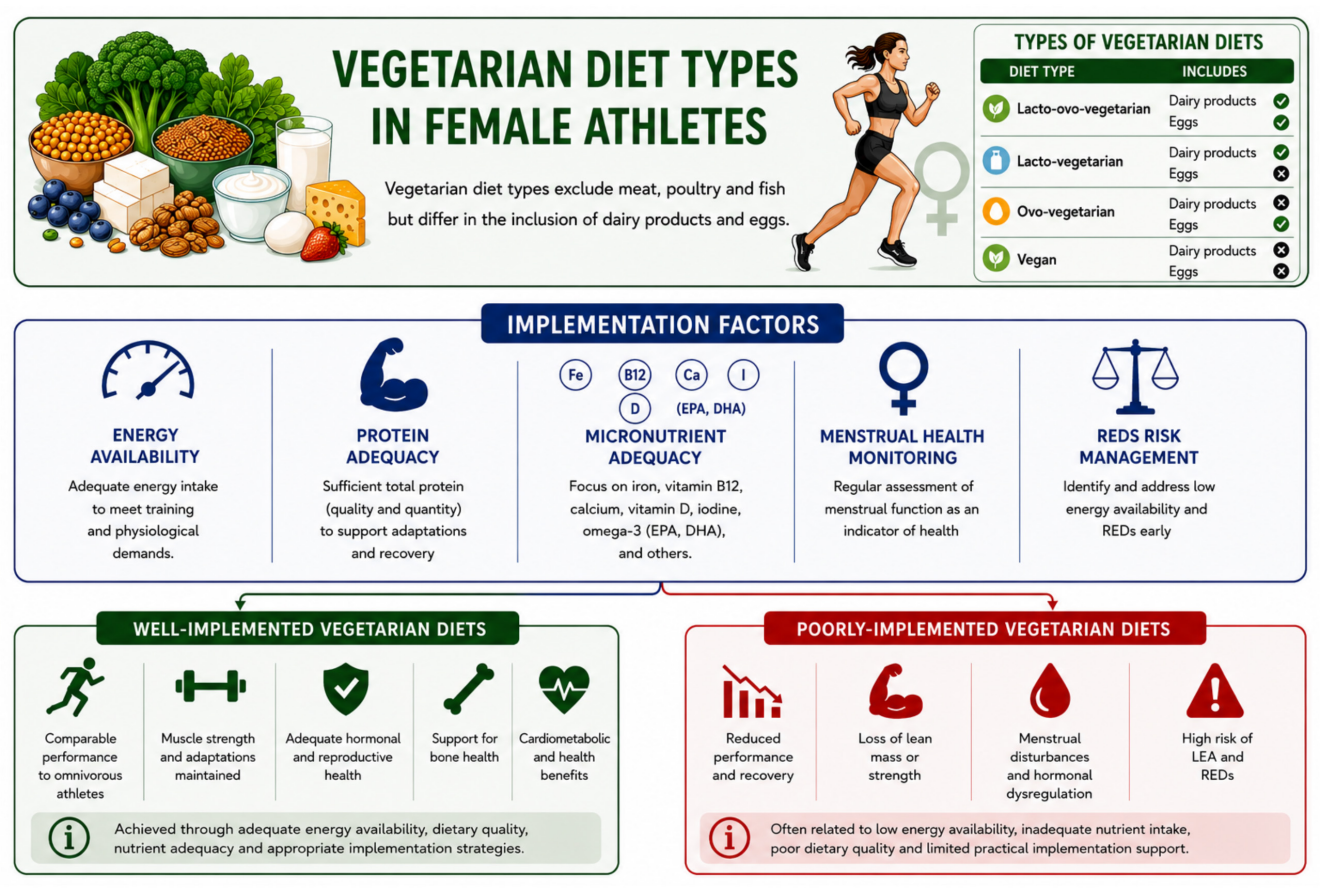
Conceptual framework illustrating the implementation gap in vegetarian diets among female athletes.

An additional challenge in interpreting the current evidence relates to the distinction between dietary efficacy and real-world implementation. Controlled interventions typically evaluate the effects of vegetarian diets under highly standardized and supervised conditions (20–22), whereas outcomes in athletic settings depend on factors such as long-term adherence, dietary quality, energy availability, and nutritional adequacy (23). Consequently, inconsistencies across studies may partly reflect differences in how these vegetarian diet types are implemented in practice. This distinction may be particularly relevant in female athletes, whose physiological outcomes are closely influenced by energy availability, nutrient intake, and reproductive function. This narrative review adopts an implementation-focused perspective, aiming to distinguish concerns related to vegetarian diets themselves from those more plausibly explained by energy availability, nutritional adequacy, dietary quality, and practical implementation in female athletes (Figure 1). Accordingly, the aim of this narrative review was to critically evaluate the evidence regarding vegetarian diets in female athletes across performance, physiological, and health-related outcomes while examining how the implementation gap may influence the interpretation of current findings and the development of practical recommendations.

Literature was identified through searches of PubMed, Web of Science, and Scopus from database inception to April 2026 using combinations of terms related to vegetarian diets, vegan diets, female athletes, sports performance, low energy availability, REDs, menstrual function, hormonal health, and dietary implementation. Additional relevant studies were identified through manual screening of reference lists. Given the narrative nature of the review, studies were selected based on their relevance to the review objectives, with priority given to intervention studies, systematic reviews, position statements, and observational studies specifically involving female athletes or reporting sex-specific outcomes. The evidence was narratively synthesized by considering study design and consistency of findings, with particular attention to implementation-related factors that may influence the interpretation of the available evidence.

## 2 Female-specific physiology

Female athletes exhibit physiological characteristics that may influence nutritional requirements, responses to dietary interventions, and health outcomes (24). Consequently, findings derived predominantly from male athletes cannot be assumed to apply directly to women, particularly when evaluating dietary strategies that may affect energy availability, substrate utilization, and endocrine function.

Fluctuations in ovarian hormones across the menstrual cycle influence exercise metabolism and substrate utilization (17). Compared with the early follicular phase, the luteal phase may be associated with greater fat oxidation and reduced carbohydrate utilization during exercise, although findings across studies remain inconsistent (25, 26). Experimental studies have also reported lower glycogen use during prolonged endurance exercise in women, suggesting a glycogen-sparing effect and a greater reliance on lipid metabolism (27, 28). These observations highlight the important role of ovarian hormones in regulating metabolic flexibility and substrate selection during exercise.

Estrogens play a central role in these metabolic adaptations. Beyond their reproductive functions, they contribute to substrate regulation, metabolic flexibility, insulin sensitivity, and recovery processes (25, 29). In women, estrogens promote lipid oxidation during exercise and are associated with greater utilization of intramyocellular triglyceride stores (30, 31). In addition, estrogens contribute to metabolic health by enhancing insulin sensitivity and maintaining skeletal muscle glucose regulation through estrogen receptor-mediated pathways, thereby supporting metabolic flexibility and efficient substrate utilization during exercise and recovery (32). Consequently, conditions associated with chronically reduced estrogen concentrations may impair metabolic flexibility, alter body composition, and negatively affect performance and recovery. This is particularly relevant in female athletes, in whom prolonged LEA spanning weeks or months can disrupt ovarian hormone production and contribute to the development of relative energy deficiency in sport (REDs). Female reproductive physiology is highly sensitive to chronic energy deficiency, with reductions in energy availability disrupting hypothalamic gonadotropin-releasing hormone secretion and ultimately suppressing ovarian estrogen production (33). Because ovarian hormones play important roles in metabolic, reproductive and skeletal function, prolonged energy deficiency may compromise multiple physiological systems and impair adaptation to training (34, 35). Therefore, the interaction between energy availability and hormonal function represents an important consideration when evaluating nutritional strategies in female athletes (36).

Collectively, these physiological characteristics highlight the complex interactions between ovarian hormonal function, energy availability, and physiological adaptation in female athletes. Consequently, nutritional interventions may not produce identical responses across sexes, and factors such as menstrual function, hormonal status and energy availability should be considered when interpreting dietary interventions in women. These considerations provide an important framework for evaluating the current evidence on vegetarian diets in female athletes.

## 3 Exercise performance, muscular adaptations and physical function

Evidence examining the effects of vegetarian diets on performance and muscle function in women remains limited and is derived primarily from short-term controlled interventions and cross-sectional studies conducted in recreationally active populations (Table 2).

**Table 2:** Characteristics and main findings of studies evaluating vegetarian diets and performance-related outcomes in women.

Study	Design	Participants	Dietary exposure	Outcomes	Main findings
Isenmann et al. (37)	Controlled trial (12 wk)	Recreationally trained women (*n* = 9)	Transition from omnivorous to vegan diet	Body composition, performance, menstrual function	Reduced protein intake was accompanied by small reductions in skeletal muscle mass, while strength and menstrual cycle parameters remained unchanged.
Martini et al. (38)	Controlled trial (16 wk RT)	Untrained women (VEG *n* = 25; NV *n* = 20)	Vegan vs. non-vegetarian diets	Muscle hypertrophy, strength, body composition	Similar gains in muscle mass and strength were observed despite lower protein intake in vegetarians.
Boutros et al. (39)	Cross-sectional	Physically active women (*n* = 56)	Vegan diet vs. omnivorous diet	Endurance performance, strength, body composition	Vegan women demonstrated comparable strength and body composition and higher endurance-related outcomes.
Nebl et al. (40)	Cross-sectional	Recreational runners (VEG *n* = 24; LOV n=26; OMN *n* = 26)	Vegan, lacto-ovo-vegetarian, or omnivorous diets	Exercise capacity, body composition	No differences in exercise capacity or body composition were observed between dietary groups.
Bartholomae et al. (42)	Cross-sectional	Lightly active women (*n* = 38)	Vegan diet vs. omnivorous diet	Protein intake, lean mass, strength	Lower protein intake in vegans was associated with lower lean mass and lower lower-body strength.

n, number of participants; LOV, lacto-ovo-vegetarian; NV, non-vegetarian; OMN, omnivorous; RT, resistance training; VEG, vegan; wk, weeks.

Isenmann et al. (37) evaluated the effects of transitioning from an omnivorous to a vegan diet in recreationally trained young women during a 12-week controlled intervention (37). Although countermovement jump performance decreased by approximately 3.3% (≈0.8 cm), the decline had already emerged before the vegan phase, preventing clear attribution to the dietary intervention. Likewise, no changes were observed in lower-body maximal strength. The vegan phase was accompanied by lower protein intake and a small reduction in skeletal muscle mass, suggesting that nutritional implementation may influence outcomes during the early stages of dietary transition. However, interpretation is limited by the sequential non-randomized design, the absence of a washout period and a small study sample (*n* = 9). In contrast, Martini et al. (38) reported comparable gains in muscle hypertrophy, lean mass, and maximal strength between vegan and non-vegetarian young women during a 16-week resistance training program (38). Participants maintained their habitual dietary patterns without protein supplementation, despite lower habitual protein intake in vegetarian participants compared with omnivores (1.0 vs. 1.3 g/kg/day). However, all participants were previously untrained women, limiting extrapolation of these findings to trained athletes or highly demanding competitive settings. Collectively, the available intervention studies suggest that both an 8-week transition from an omnivorous to a vegan diet and longer-term adherence to a vegan diet do not appear to impair strength or performance outcomes in women. Although the transition to a vegan diet was accompanied by lower protein intake and modest reductions in skeletal muscle mass, these changes did not translate into impaired strength outcomes. Similarly, the available study in women suggests that long-term adherence to a vegan diet can support comparable muscular adaptations to resistance training under controlled conditions (38).

Cross-sectional studies conducted under free-living conditions have generally reported comparable muscle-related outcomes between vegan and omnivorous women across populations with different training status and habitual dietary patterns. Boutros et al. (39), in physically active women engaged primarily in aerobic activities, observed similar lean body mass (41.8 vs. 41.5 kg) and lower-body muscle strength (2.4 vs. 2.5 kg/kg lean body mass) between vegan and omnivorous participants. Although vegan participants exhibited higher estimated VO_2_max values (44.5 vs. 41.6 ml/kg/min) and longer submaximal endurance time to exhaustion (12.2 vs. 8.8 min), these findings should be interpreted cautiously given the cross-sectional design and the recreationally active nature of the study population (39). Likewise, Nebl et al. reported similar maximal exercise capacity among vegan, lacto-ovo-vegetarian and omnivorous recreational runners in sex-stratified analyses, with no differences in maximal power output relative to body weight (women: 3.99–4.06 W/kg; men: 4.39–4.43 W/kg) (40). Similar findings were also reported by Schemes et al., who evaluated mostly resistance-untrained women with low recent exercise exposure and found no differences in muscle size, lower-body strength or countermovement jump performance despite lower protein intake among vegan participants (41). In contrast, Bartholomae et al. reported less favorable outcomes in lightly active young women following vegan diets compared with omnivores (42). Several lower-body strength measures were 14%−15% lower in vegan participants, accompanied by lower total lean mass and a higher proportion of participants not achieving the recommended protein intake of 0.8 g/kg/day (58 vs. 21%). Notably, physical activity levels were also substantially lower in the vegan group, making it difficult to determine whether the observed differences were attributable to dietary pattern itself or to broader lifestyle and behavioral factors. Additionally, dietary intake was assessed using a single 24-h recall, and the relatively small sample size may have increased the risk of measurement bias and residual confounding. Taken together, the available cross-sectional evidence generally supports the notion that long-term adherence to vegetarian diets is not necessarily associated with impaired muscle function or exercise capacity in women under real-world conditions. However, interpretation remains limited by the observational nature of these studies, the heterogeneity of the populations evaluated, and the possibility of long-term adaptation and selection biases.

Overall, the available evidence suggests that vegetarian diets are not inherently associated with impaired muscle function or exercise capacity in women. However, the limited number of controlled studies, the predominance of observational evidence, and the heterogeneity of the populations evaluated preclude definitive conclusions, particularly in trained athletes and during short-term dietary transition phases. Importantly, some of the variability observed across studies may reflect differences in dietary implementation, including protein intake, energy availability, training status, and duration of dietary adherence. Consequently, interpretation of the current evidence requires consideration of these factors alongside the dietary pattern itself.

## 4 Nutritional profile of vegetarian diets in athletes

Vegetarian diets present a distinct nutritional profile compared with omnivorous diets, characterized by differences in macronutrient composition, micronutrient intake and bioactive compounds. Some of these characteristics may be especially relevant in female athletes due to the interaction between nutritional status, energy availability, and reproductive physiology. Although vegetarian diets share common principles, important nutritional differences exist between lacto-ovo-vegetarian and vegan diets owing to the degree of animal-food exclusion. In general, vegan diets tend to provide higher fiber and lower energy density, whereas lacto-ovo-vegetarian diets retain dairy products and eggs, which contribute additional protein, fat and selected micronutrients (1).

### 4.1 Energy intake

Adequate energy intake is a key determinant of athletic performance and physiological function (43). Vegetarian diets are characterized by a higher fiber content, lower energy density, and greater food volume, which may make it more challenging for athletes with high energy expenditure to meet their energy requirements (44, 45). This issue may be particularly relevant in female athletes during periods of intensified training or intentional body mass reduction, when the likelihood of LEA may be greater (46, 47). Importantly, these challenges are not inherent limitations of vegetarian diets and can generally be addressed through appropriate dietary planning and food selection strategies (48). These characteristics are generally more pronounced in vegan than in lacto-ovo-vegetarian diets, owing to the greater fiber content and lower energy density of whole-foods vegan diet. Consequently, athletes transitioning to a vegan diet may require additional nutritional guidance to ensure adequate energy intake (49, 50).

### 4.2 Carbohydrates and fiber

Carbohydrates are the main energy source during moderate- and high-intensity exercise and play a key role in glycogen replenishment and recovery (51). However, female athletes may implement intentional carbohydrate restriction driven by concerns regarding body weight and composition (52), which impairs glycogen replenishment, recovery and training adaptation. In this context, well-planned vegetarian diets may offer a nutritional advantage, as they include greater amounts of carbohydrate-rich foods such as fruits, vegetables, whole grains, and legumes (14).

Vegetarian diets also generally provide higher amounts of dietary fiber, a nutritional component that has historically received limited attention in sports nutrition. Emerging evidence suggests that fiber may influence athletic health and performance through effects on gastrointestinal function, gut microbiota composition, and systemic inflammation (53). Although higher fiber intakes may support gut and cardiometabolic health, excessive intake may also increase gastrointestinal discomfort in some athletes, particularly around competitions or intense training sessions (54). Dietary planning should aim to balance the potential health benefits of fiber with gastrointestinal tolerance and the practical demands of training and competition.

### 4.3 Protein

Differences in protein adequacy may represent an important implementation-related factor when evaluating muscle-related outcomes in athletes following vegetarian diets. Although nitrogen balance studies found no statistically significant effect of sex on protein requirements in the general population (55), protein adequacy may be particularly relevant in female athletes due to the combined demands of training, recovery and, in some cases, lower habitual energy intake (56). Plant proteins generally differ from animal proteins in digestibility and amino acid composition, including a lower content of certain essential amino acids such as leucine, a key regulator of muscle protein synthesis (57). Despite these differences, current evidence suggests that, when total protein intake is sufficient and a variety of complementary plant protein sources are consumed throughout the day, well-planned vegetarian diets can provide a complete essential amino acid profile and support comparable adaptations in muscle strength, lean mass and training outcomes (58, 59). Emerging evidence also suggests that women may have lower leucine requirements to maximally stimulate muscle protein synthesis than those derived from male-based studies, possibly due to estrogen-mediated lower leucine oxidation and greater muscle anabolic sensitivity, although this remains an evolving area of research (60). Though some studies have reported lower muscle mass and strength among individuals following vegan diets (37, 42), these findings should be interpreted cautiously, as many studies are limited by small sample sizes, cross-sectional designs and inadequate total protein intake.

Creatine may represent another relevant nutritional consideration for female athletes following vegetarian diets. Because creatine is obtained almost exclusively from animal-source foods, individuals following vegetarian and especially vegan diets generally exhibit lower circulating and intramuscular creatine stores than omnivores, although the functional implications of these differences remain uncertain (61). A 6-month randomized controlled trial in omnivorous women switching to a lacto-ovo-vegetarian diet found that muscle total creatine declined by 14.6% after 3 months (from 153.5 to 128.8 mmol/kg dry weight), whereas plasma creatine decreased by 46%. These reductions were prevented by supplementation with 1 g/day of creatine monohydrate (62). Consistent with these findings, a systematic review concluded that creatine supplementation reliably increases muscle creatine stores in vegetarians and may enhance lean mass, muscular strength, and high-intensity exercise performance. However, whether vegetarian individuals derive greater ergogenic benefits than omnivores remains inconsistent across studies, and evidence specifically involving female athletes is still scarce (63). Therefore, although routine creatine supplementation cannot currently be recommended for all female athletes following vegetarian diets, it may represent a relevant consideration for those participating in sports requiring repeated high-intensity efforts or resistance training.

### 4.4 Bioactive compounds

Vegetarian diets are typically rich in bioactive compounds, including antioxidants and dietary nitrates, due to their high content of fruits, vegetables and legumes (64). Antioxidants such as vitamin C, vitamin E, carotenoids and polyphenols may help attenuate exercise-induced oxidative stress and support recovery and immune function (65). This may be relevant in female athletes, particularly under conditions of high training loads, LEA, or menstrual dysfunction (18). However, the effects of antioxidant-rich diets on exercise adaptations remain context-dependent, as exercise-induced reactive oxygen species are not only involved in oxidative damage but also act as signaling molecules for training adaptation (66). Consequently, the impact of dietary antioxidants depends on factors such as their source, dosage, timing relative to exercise, training modality and status, and overall dietary context (67).

Dietary nitrates, abundant in vegetables such as beetroot, spinach and arugula, contribute to nitric oxide production and may improve vasodilation, muscle perfusion and oxygen delivery during exercise (68). Higher nitrate intake has been associated with improved muscle efficiency and reduced oxygen cost during exercise, particularly in endurance and high-intensity intermittent activities, although responses appear to vary according to training status and exercise modality (69).

Although these compounds may provide physiological advantages, their contribution is unlikely to compensate for insufficient energy availability or nutritional inadequacy, reinforcing the importance of overall dietary implementation.

### 4.5 Micronutrients

Vitamin B12 is a water-soluble vitamin of bacterial origin that is not naturally present in plant foods in its active form (70). Therefore, individuals following vegetarian diets require supplementation or regular consumption of fortified foods. Inadequate intake may lead to deficiency, potentially impairing performance through fatigue and altered energy metabolism (5, 71).

Iron from plant foods is predominantly non-haem iron, which has lower bioavailability than haem iron from animal-derived foods. Nevertheless, individuals following vegetarian diets may develop adaptive mechanisms that improve iron absorption over time (72). Iron is particularly relevant for athletes because of its role in oxygen transport and energy metabolism (73, 74). Female athletes may be especially vulnerable to iron deficiency due to the combined effects of menstrual blood losses, exercise-related iron losses and, in some cases, insufficient dietary intake or reduced iron bioavailability (75). This issue may be particularly relevant in endurance disciplines and under conditions of LEA, where inadequate iron status may impair aerobic performance, recovery, and overall physiological function.

Calcium and vitamin D intake may also warrant particular attention in female athletes due to their critical role in bone and muscular health (5). This may be especially relevant under conditions of LEA, which have been associated with impaired bone metabolism and increased risk of bone stress injuries (76).

## 5 Energy availability and REDs risk

Energy availability is defined as the amount of dietary energy remaining to support physiological functions after accounting for exercise energy expenditure, relative to fat-free mass (18, 36). LEA is the primary pathophysiological mechanism underlying REDs, a syndrome associated with endocrine disturbances, menstrual dysfunction, impaired bone health, stress fractures, fatigue and impaired training adaptation (77, 78). Female athletes may be particularly vulnerable to LEA and REDs because of the interaction between high energy expenditure, reproductive physiology and body composition pressures, particularly in endurance and aesthetic sports (19). These considerations are especially relevant when evaluating dietary patterns that may influence energy intake and nutritional adequacy.

The relationship between vegetarian diets and REDs has received increasing attention because several characteristics of these dietary patterns may theoretically influence energy availability in female athletes. Some vegetarian diets, particularly vegan diets, may be characterized by lower energy density and higher fiber intake (79), factors that may increase satiety and reduce spontaneous caloric intake (80, 81). This could be particularly relevant in athletes with high physiological demands or pre-existing risk of LEA, especially during periods of intensified training or intentional body mass reduction. However, current evidence does not consistently support a direct association between vegetarian diets and prolonged LEA, and several studies have demonstrated comparable body composition and physiological outcomes between vegan and omnivorous athletes when energy intake is appropriately planned (39, 58). Moreover, vegetarian diets are often associated with higher carbohydrate and antioxidant intakes, characteristics that may support glycogen availability, recovery and endurance performance (5, 80). An additional concern is that vegetarian diets may occasionally be adopted to conceal eating disorders, particularly in sports emphasizing leanness or body weight (82). However, these dietary patterns are not inherently associated with pathological eating behaviors, and the presence of an ED depends on an individual's motivations and the broader clinical context, rather than the dietary pattern itself (83).

Within the context of REDs, protein adequacy and bone health represent two key nutritional considerations for female athletes following vegetarian diets. Several studies have described lower intakes of protein, leucine, calcium, and vitamin D in vegan athletes, particularly when dietary planning and implementation are suboptimal (5, 36). In the presence of prolonged LEA, these nutritional factors may further compromise muscle recovery, endocrine function, and skeletal health. Nevertheless, systematic reviews and meta-analyses indicate that vegetarian diets can provide sufficient protein and micronutrients and do not appear to impair muscular strength or bone health when nutritional requirements are achieved (58, 84). Similarly, despite differences in iron bioavailability, current evidence does not consistently indicate poorer iron status among vegetarian compared with omnivorous athletes (85, 86).

Overall, current evidence does not support vegetarian diets as an independent risk factor for REDs in female athletes. Rather, current evidence suggests that REDs risk is more closely related to energy availability and nutritional adequacy than to whether athletes follow a vegetarian or omnivorous diet.

## 6 Menstrual function and hormonal health

Concerns have been raised regarding the potential effects of vegetarian diets on hormonal health and menstrual function in women. Particular attention has focused on soy-derived phytoestrogens because of their structural similarity to endogenous estrogens and their widespread consumption among individuals following vegetarian diets (87). In addition, indirect effects mediated through energy availability and body composition have also been proposed as potential mechanisms influencing reproductive function (88).

Despite these theoretical concerns, empirical evidence supporting a detrimental effect of vegetarian diets on hormonal health remains scarce. To date, the only intervention study, conducted in young recreationally trained women transitioning from an omnivorous to a vegan diet, reported no adverse effects on menstrual cycle characteristics or salivary hormonal measures after 8 weeks, despite reductions in relative protein intake (1.39–1.06 g/kg/day), body weight (68.2–67.7 kg), and skeletal muscle mass (29.4–28.7 kg), highlighting the potential importance of dietary implementation during the transition phase (89). Observational studies have reported differences in hormonal biomarkers between dietary groups, including higher concentrations of sex hormone-binding globulin (SHBG) and lower levels of free estradiol, free testosterone, and Dehydroepiandrosterone sulfate (DHEA-S) among vegetarian women (90). However, the clinical significance of these findings remains uncertain. Furthermore, the observational nature of these studies prevents distinguishing dietary effects from potential confounding influences such as body composition, physical activity or other lifestyle factors. Therefore, current evidence does not indicate that vegetarian diets adversely affect hormonal health, although robust long-term studies are lacking.

Soy-derived phytoestrogens have received considerable attention due to their structural similarity to endogenous estrogens and their widespread consumption among individuals following vegetarian diets (91). However, current evidence provides limited support for clinically relevant adverse effects on menstrual or hormonal health. Although some studies have reported modest associations between phytoestrogen intake and menstrual cycle characteristics, the observed effects have generally been small and of uncertain clinical significance (92). Furthermore, a recent systematic review in women with polyendocrine metabolic ovary syndrome reported favorable effects of phytoestrogens on the luteinizing hormone (LH)/follicle-stimulating hormone (FSH) ratio, SHBG concentrations and insulin sensitivity (93). Although phytoestrogens may exert measurable effects on endocrine biomarkers, current evidence does not support clinically meaningful adverse effects on menstrual function or hormonal health (94).

Taken together, the available evidence suggests that concerns regarding menstrual dysfunction in female athletes following vegetarian diets are more likely to relate to energy availability and nutritional adequacy than to vegetarian eating *per se*.

## 7 Future directions and methodological challenges

The findings of this narrative review should be interpreted in light of several methodological limitations. First, as a narrative review, it did not follow a formal systematic review methodology; therefore, study identification and selection were not based on predefined systematic procedures. Second, no formal risk-of-bias assessment was performed, limiting the ability to weigh the relative strength of individual findings. Third, the broad scope of the review, encompassing exercise performance, muscular adaptations, hormonal health, menstrual function and REDs-related outcomes, necessarily limits the depth with which each topic can be addressed. Furthermore, the review includes evidence from populations with markedly different training status, ranging from physically active women to recreational and competitive athletes. This heterogeneity may limit the generalizability of the findings, as physiological demands and nutritional requirements differ substantially according to athletic level and sport participation. Finally, this review encompasses both lacto-ovo-vegetarian and vegan diet types, which, despite sharing the common principle of excluding meat and meat-derived foods, differ in several nutritional characteristics and practical considerations. Therefore, some conclusions may not apply equally across all vegetarian diet types.

These limitations should be interpreted alongside the broader limitations of the available evidence. First, women continue to be underrepresented in sports nutrition research, and many recommendations regarding vegetarian diets are still derived from studies conducted predominantly in men. Given the well-established sex-specific differences in substrate utilization, endocrine responses, energy metabolism and reproductive physiology, extrapolation of findings from male cohorts may not adequately reflect the needs and responses of female athletes.

A second major limitation relates to the inadequate control of female-specific physiological variables. Many studies fail to account for menstrual cycle phase, hormonal contraceptive use, menstrual status or menopausal status, despite the potential influence of these factors on metabolism, exercise performance, recovery and endocrine outcomes (95). Improved characterization and reporting of these variables should be considered a priority in future investigations involving female athletes.

The current evidence base is also dominated by cross-sectional studies and short-term interventions. While these designs provide valuable preliminary information, they are limited in their ability to establish causality or evaluate long-term adaptations to vegetarian diets. Longitudinal studies and randomized controlled trials are therefore needed to determine whether the effects observed during short-term dietary transitions persist over time and to better characterize adaptations occurring under real-world conditions. An additional challenge relates to the limited characterization of dietary implementation across studies. Factors such as energy availability, dietary quality, and duration of adherence are often poorly reported, making it difficult to distinguish the effects of vegetarian diets from the effects of their implementation.

Finally, future research should move beyond traditional physiological outcomes such as VO_2_max and body composition to include outcomes that are particularly relevant to female athletes, including menstrual function, endocrine health, bone health, injury risk, recovery, training adaptation and indicators of LEA (95). Such approaches may help clarify whether vegetarian diets exert meaningful effects on health and performance or whether observed differences are primarily explained by variations in dietary implementation (96).

Addressing these limitations will be essential to distinguish the physiological effects of vegetarian diets from the effects of their implementation, thereby helping to close the current implementation gap and develop evidence-based recommendations specifically tailored to female athletes.

## 8 Practical applications

The practical implications of this review emphasize that bridging the implementation gap requires moving the focus from dietary classification to the quality of dietary implementation. Accordingly, nutritional support should prioritize identifying and addressing implementation-related factors, including energy availability, dietary quality, and nutritional adequacy, rather than focusing solely on whether an athlete follows a vegetarian or an omnivorous diets.

### 8.1 Prioritize energy availability

Current evidence suggests that the primary concern when implementing vegetarian diets in female athletes is not the exclusion of animal-source foods *per se*, but the achievement of adequate energy availability. Particular attention may be warranted during periods of intensified training, intentional body mass reduction, or highly competitive demands (39, 58).

### 8.2 Assess dietary adequacy beyond dietary pattern

Classification of athletes as vegan, vegetarian, or omnivorous provides limited information regarding nutritional adequacy. Practitioners should therefore evaluate energy intake, protein intake, micronutrient status, and overall dietary quality rather than relying solely on dietary labels when assessing nutritional risk.

### 8.3 Monitor female-specific outcomes

Given the potential interaction between energy availability, endocrine function and reproductive health, routine monitoring of menstrual function, body composition and indicators of REDs may provide valuable information regarding the adequacy of dietary implementation (95).

### 8.4 Ensure nutritional adequacy

Although well-planned vegetarian diets can meet the nutritional demands of female athletes, particular attention may be required for nutrients commonly identified as nutritional considerations in vegetarian populations, including vitamin B12, iron, calcium, vitamin D, and long-chain omega-3 fatty acids (80). Because preformed long-chain omega-3 fatty acids eicosapentaenoic acid (EPA) and docosahexaenoic acid (DHA) are largely absent from vegetarian and especially vegan diets, algae-derived EPA and DHA supplements may be considered when dietary intake is insufficient, although evidence supporting performance-related benefits in female athletes remains limited (5, 97).

### 8.5 Focus on implementation rather than dietary labels

Current evidence suggests that performance, health, and REDs-related outcomes are more closely associated with energy availability, protein adequacy, and overall dietary quality than with the exclusion of animal-source foods. Consequently, practitioners should focus on how vegetarian diets are implemented rather than whether athletes identify as vegan or vegetarian.

Addressing the implementation gap requires moving beyond nutrient-based recommendations toward practical nutrition education and individualized dietary support. Female athletes should be empowered to understand the nutritional principles underlying well-planned vegetarian diets, enabling them to adapt food choices to changing training loads, competition schedules and body composition goals while maintaining adequate energy availability and nutrient intake. This process should be supported by coaches, families and sports nutrition professionals, particularly in adolescent and young adult athletes, to facilitate sustainable dietary habits and early identification of nutritional challenges. Recent evidence also suggests that knowledge gaps regarding plant-based nutrition remain among healthcare professionals, highlighting the need for further education and training in this area (98, 99).

Rather than representing a single dietary decision, successful implementation should be viewed as an ongoing process requiring structured nutritional management throughout the athletic season. From a practical perspective, this process should include an initial assessment of nutritional status and REDs risk, individualized dietary planning to ensure adequate energy and nutrient intake, practical nutrition education to promote autonomous food selection, and periodic monitoring of dietary adherence, menstrual function, training adaptation, and relevant biomarkers when indicated. Such an implementation-oriented approach may help distinguish challenges related to dietary execution from those attributable to the dietary pattern itself. Ultimately, successful implementation depends not only on the dietary pattern itself but also on the athlete's nutritional knowledge, the expertise of the multidisciplinary team, and the use of structured implementation strategies that facilitate long-term dietary adherence and nutritional adequacy.

## 9 Conclusion

Current evidence does not indicate that vegetarian diets inherently compromise performance, muscle adaptations, hormonal health or increase REDs risk in female athletes. Although the evidence base remains limited, many of the concerns commonly attributed to vegetarian diets appear to be more closely related to energy availability, nutritional adequacy, and overall dietary implementation than to the exclusion of animal-source foods *per se*. Consequently, interpretation of the current literature should consider how vegetarian diets are implemented rather than focusing solely on dietary classification. This implementation-focused perspective may help explain inconsistencies in the current literature and provide a more useful framework for practitioners working with female athletes following vegetarian or vegan diets. Future studies should also evaluate implementation strategies aimed at facilitating the adoption and long-term maintenance of well-planned vegetarian diets in athletic populations, including nutrition education, behavioral support, and multidisciplinary care models.
